# Quantitative Characterization of the Hemorrhagic, Necrotic, Coagulation-Altering Properties and Edema-Forming Effects of Zebra Snake (*Naja nigricincta nigricincta*) Venom

**DOI:** 10.1155/2018/6940798

**Published:** 2018-10-24

**Authors:** Erick Kandiwa, Borden Mushonga, Alaster Samkange, Ezequiel Fabiano

**Affiliations:** ^1^School of Veterinary Medicine, Faculty of Agriculture and Natural Resources, Neudamm Campus, University of Namibia, P. Bag 13301, Pioneers Park, Windhoek, Namibia; ^2^Department of Wildlife Management and Ecotourism, Katima Mulilo Campus, Faculty of Agriculture and Natural Resources, University of Namibia, P. Bag 1096, Ngweze, Katima Mulilo, Namibia

## Abstract

This study was designed to investigate the cytotoxicity and haemotoxicity of the Western barred (zebra) spitting cobra (*Naja nigricincta nigricincta*) venom to help explain atypical and inconsistent reports on syndromes by Namibian physicians treating victims of human ophidian accidents. Freeze-dried venom milked from adult zebra snakes was dissolved in phosphate buffered saline (PBS) for use in this study. Haemorrhagic and necrotic activity of venom were studied in New Zealand albino rabbits. Oedema-forming activity was investigated in 10-day-old Cobb500 broiler chicks. Procoagulant and thrombolytic activity was investigated in adult Kalahari red goat blood* in vitro*. The rabbit skin minimum hemorrhagic dose (MHD) for* N. n. nigricincta* was 9.8*μ*g. The minimum necrotizing dose (MND) for* N. n. nigricincta* venom was 12.2*μ*g. The* N. n. nigricincta* venom showed linear dose-dependent procoagulant activity on goat blood (p<0.05). Likewise,* N. n. nigricincta* venom showed linear dose-dependent thrombolytic activity on goat blood (p<0.05, n = 6). Subplantar injection of* N. n. nigricincta* venom (25*μ*g, 50*μ*g, 75*μ*g, and 100*μ*g) into chick paw resulted in peak oedema of 35.5%, 38.5%, 42.9%, and 47.5%, respectively, two hours after injection. Paw oedema subsided within five hours to a mean volume ranging from 5% (25*μ*g venom) to 17.6% (100*μ*g venom). In conclusion, though* N. n. nigricincta* belongs to the genus* Elapidae*, the current study has shown its venom to possess potent hemorrhagic, necrotic (cytotoxic), and paradoxically, both procoagulant and thrombolytic activity. The authors propose further work to fractionate, isolate, and elucidate the structure of the various* N. n. nigricincta* venom toxins as a prelude to the development of an antivenom.

## 1. Introduction

Since time immemorial, man has always suffered from envenomation resulting from snakebites. Although accurate statistics have proved elusive, it is estimated that the global burden of snakebites stands at 1.2 to 5.5 million bites per year, 25,000-125,000 deaths per year, and about 400,000 victims left with permanent disability [1]. In 2009, snakebite was declared a neglected tropical disease by the WHO [2]. The World Health Organization declared snake envenomation as a significant Sub-Saharan disease problem [3].

In Namibia, like in most other developing countries, the majority of snake bites result from the overlap of human and snake habitats, domiciliation of rodents (main prey of most snakes), the nocturnal and heat seeking poikilothermic nature of snakes, and accidents during snake handling. Some of these snakebites lead to fatalities and wound complications culminating in debilitating physical deformities in victims [4, 5] and associated socioeconomic problems resulting from these disabilities [6, 7]. The vast size of Namibia as a country also poses a potential problem of bringing emergency health care to such snakebite victims.

Venomous snakes belong to five main families:* Hydrophiinae, Elapidae, Viperidae*,* Crotalidae,* and* Colubridae* [8, 9]. These snakes possess venom glands that can synthesize, store, and secrete up to 50-60 proteins/peptides of varying structure but are capable of causing damage at the bite site and systemically [2, 10]. The venom components are usually fairly similar in snakes of the same family [1]. Venoms of snakes belonging to the families* Elapidae *(mainly cobras and mambas) and* Hydrophiinae* (mainly sea snakes) are highly neurotoxic and produce flaccid paralysis and respiratory paralysis in animals [2, 11–15].* Viperidae* (vipers),* Colubridae* (back-fanged venomous snakes, e.g., Boomslang and the Twig snake), and* Crotalidae* (pit-vipers) venoms produce in addition to systemic/lethal effects, striking local effects, namely, hemorrhage, necrosis, and oedema [11, 16] as well as alterations in coagulability of blood [17–19]. The protein components of the spitting cobra of* Naja sputatrix* comprises the proteins, three-finger toxins (3FTXs), phospholipase A_2_ (PLA_2_), nerve growth factors, and snake venom metalloproteinase in that order [15]. The Zebra snake (*N. n. nigricincta*) is a venomous spitting snake belonging to the* Elapidae* family and found only in Namibia and Southern Angola [20].

Though belonging to the family of Elapids, empirical evidence suggests that the Zebra snake has acquired highly potent cytotoxic, hemorrhagic, anticoagulant, and thrombolytic toxins whilst retaining their familial neurotoxins. Namibia has had a very high number of both human and animal victims of the Zebra snake (Buys, 2016; personal communication). Snake antivenom immunoglobulins are the only specific treatment for envenoming by snakebites [4, 21]. Clinically, administering antivenom to the affected patient within a very limited time frame (<2 hours) efficiently reverses many of the detrimental systemic effects caused by snake venom [22]. South African Institute for Medical Research (SAIMR) polyvalent antivenom that is currently used in Namibia at a cost of almost US$100.00 per vial is obviously not affordable to the average rural dweller. This polyvalent antivenom was developed against venoms from puff adder, gaboon viper, rinkals, mambas, cape cobra, forest cobra, snouted cobra, and Mozambique spitting cobra but not the zebra snake.

The efficacy of SAIMR polyvalent antivenom against* N. n. nigricincta *envenomation has reportedly not been satisfactory as specific treatment for this medically important and almost exclusively Namibian snake (Buys 2016, personal communication). Though fatalities are reportedly low, Namibian physicians have resorted to fasciotomy/debridement of necrotic lesions [23] followed by skin grafting due to the fact that in this vast country, victims often fail to receive this polyvalent antivenom within the postulated 2-hr window after bite (Buys 2016, personal communication). The aim of this study was to quantitatively characterize the cytotoxic (necrotic), hemorrhagic, procoagulant, thrombolytic, and oedema-forming effects of the Namibian zebra snake venom using WHO approved protocols [24].

## 2. Materials and Methods

### 2.1. Snake Venom

Venom was carefully and humanely milked by an expert snake handler from snakes that were caught in suburban Windhoek and later relocated to the surrounding Savanna bushveld after replenishment of venom gland stores. Approximate age, sex, length, girth, location of capture, and location of release were geo-referenced and recorded on a database for surveillance of snakes in and around Windhoek. Venom was diluted with distilled water and freeze-dried overnight using a Vitis Freeze Dryer (United Scientific). The resultant venom powder from each snake was stored in a separate sealed and appropriately labelled glass vial at -30°C until time of use. For this study, serial dilutions of venom (250 to 1000*µ*g/ml) from a single snake were made by dissolving known quantities of venom powder in Phosphate Buffered Saline at pH 7.4 freshly prepared from tablets (Sigma-Aldrich). Sterile 0.5ml needles were used to administer intradermal and subplantar injections of solutions.

### 2.2. Animals

Male and female albino New Zealand rabbits (about 2.5 kg weight) were obtained through City Pets, Windhoek, and reared in the small stock section at Neudamm farm, University of Namibia. Day old broiler Cobb500 chicks were obtained from Namib Poultry and reared in the Poultry section at Neudamm farm. Blood for thrombolytic studies was obtained from Kalahari red stud goats reared at the Neudamm farm.

### 2.3. Minimum Hemorrhagic Dose (MHD)

The MHD is defined as the least amount of venom (*μ*g dry weight) which, when injected intradermally into rabbits, results in a hemorrhagic lesion of 10 mm diameter 24 hours later [25]. Aliquots of 0.1 ml PBS containing 7.5, 10, 25, 50, 75, and 100*μ*g of venom were injected into the shaved dorsal skin of each of six adult rabbits marked with grids of 25mm squares (n=6). Three replicates were performed for each dose on different randomly chosen squares of the grid on each rabbit and then mean values were determined for each concentration on each animal. The animals were sacrificed after 24 hours, the dorsal skin was removed, and the diameter of the lesions was measured on the inner surface of the skin in two directions at right angles using calipers and with the aid of background illumination. The MHD was calculated using the regression equations relating the doses of venom to the mean diameters of the haemorrhagic lesions.

### 2.4. Minimum Necrotic Dose (MND)

The MND is defined as the least amount of venom (*μ*g dry weight) which, when injected intradermally into rabbits, results in a necrotic lesion of 5mm diameter 72 hours later [25]. The method used was the same as that for the MHD, except that the skin was removed 72 hours after injection. The MND was calculated using the regression equations relating the doses of venom to the mean diameters of the necrotic lesions.

### 2.5. Percentage Thrombolysis

Venous blood was drawn from healthy adult male Kalahari red goats (n = 6) of which 500*μ*L of blood was transferred to each of previously weighed microcentrifuge tubes to form clot. Phosphate buffered saline (PBS) at pH 7.4 was added to lyophilized heparin vial (1000 I.U.) and mixed properly to create a stock solution from which serial dilutions of 0.05, 0.5, 5, and 50 I.U. heparin were made for observation of thrombolytic activity of heparin using the* in vitro* method developed by Prasad* et al*., 2006. This protocol was adapted to measure the thrombolytic activity of 100*μ*L solutions containing 25, 50, 75, or 100 *µ*g Zebra snake venom on 500*μ*L goat blood. This experiment was repeated three times with blood from each animal and mean values were determined for each concentration for blood from each animal.

### 2.6. Coagulation-Altering Activity

Venous blood was drawn from healthy adult male Kalahari red goats (n = 6) of which 500*μ*L of blood was transferred to each of previously weighed 1.5ml Eppendorf tubes containing 100*μ*L of 250, 500, 750, or 1000*μ*g/ml of venom, 0.05, 0.5, 5, or 50 I.U. heparin (positive control) or 100*μ*L of PBS (negative control) and gently mixed to avoid haemolyzing the blood. The mixtures were incubated at 37°C for 90 minutes to allow clots to form. After the incubation period, filter paper strips were used to drain any liquid contents from the microcentrifuge tubes. This experiment was repeated three times and mean values were determined for each concentration for blood from each animal. The clot weight was then determined and compared with mean clot weight from tubes mixed with PBS. Percentage coagulation was calculated using the equation below:

(1) %Coagulation=clot  weight  in  tube  with  venom  or  heparinclot  weight  in  tube  with  PBS×100

### 2.7. Edema-Forming Effects

Subplantar injection of known quantities (25, 50, 75, and 100*μ*g in 0.1ml PBS solution) of snake venom was performed into the right paws of 10-day-old chicks (about 250 - 300g weight). Eighteen chicks were used for this protocol. The change in the paw volume was quantified using the chick paw edema method by Fereidoni and coworkers [26] and improved by Ainooson and others [27]. Formalin (2.5%) was used as a standard edema-forming substance (positive control) and PBS was used as the negative control. The experiment was repeated three times using different chicks for each level of treatment and mean values of proportional change in paw size per concentration were determined.

### 2.8. Ethical Statement

All animals were used with the ethical approval from the University of Namibia Ethical Clearance Committee (Certificate: NCREC/01/2018/1). All procedures performed on animals and disposal of animals/animal tissues followed a protocol approved by the University of Namibia Ethical Clearance Committee. In the course of this study, the researchers strictly adhered to the WHO guidelines [24].

### 2.9. Statistical Analysis

Descriptive and inferential statistics were performed in SPSS version 25 using one way ANOVA with Tukey's post-hoc test. P values ≤0.05 were considered statistical significant.

## 3. Results

**Figure 1:**
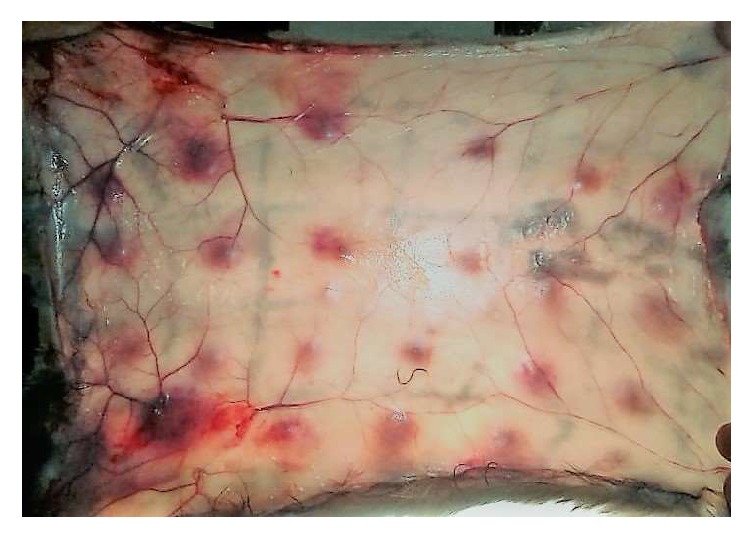
Haemorrhagic lesions on internal aspect of rabbit skin 24 hours after intradermal injection of varying doses of* N. n. nigricincta* venom.

As shown in Figure 1, intradermal injection of* N. n. nigricincta* venom produced significant haemorrhagic lesions within 24hrs of injection. Maximum average diameter (40mm, n = 6) was recorded with the highest amount of* N. n. nigricincta* venom (100*µ*g) injected.

**Figure 2:**
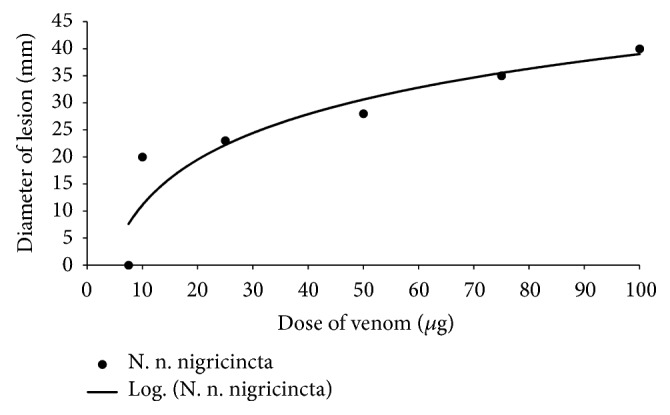
The dose-dependent hemorrhagic activity of* N. n. nigricincta *venom on adult rabbit skin.

As shown in Figure 2,* N. n. nigricincta* venom showed a significant dose-dependent increase in the diameter of the hemorrhagic lesions with each increase in the amount of venom injected into each site (p<0.05, n = 6). Hemorrhagic lesion diameter showed a very strong logarithmic dependence on dose of venom injected (R^2^ = 0.90). The MHD determined from this relationship for* N. n. nigricincta* was 9.8*μ*g.

**Figure 3:**
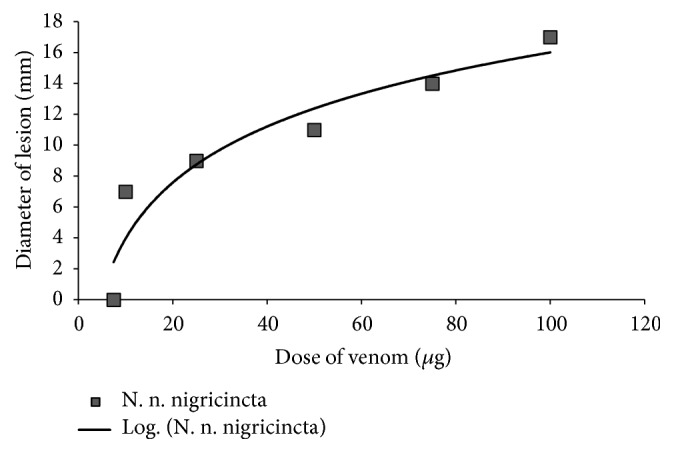
The dose-dependent necrotic activity of* N. n. nigricincta *venom on adult rabbit skin.

As shown in Figure 3,* N. n. nigricincta* venom showed a significant dose-dependent increase in the diameter of the necrotic lesions with each increase in the amount of venom injected into each site (p<0.05, n = 6). Necrotic lesion diameter showed a very strong logarithmic dependence on dose of venom injected (R^2^ = 0.93). The MND was determined from this relationship for* N. n. nigricincta* (12.4 *μ*g).

**Figure 4:**
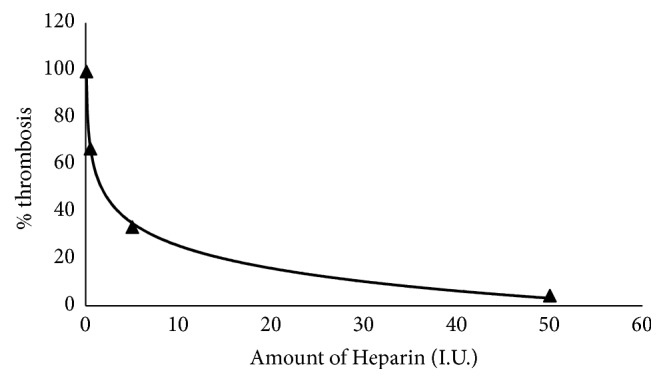
The dose-dependent anticoagulative activity of heparin on Kalahari red goat blood.

Percentage thrombosis of goat blood showed an almost perfect negative logarithmic dependence on the dose of heparin (R^2^ = 0.9991) (Figure 4). Each increase in amount of heparin (0.05 I.U., 0.5 I.U., 5 I.U., and 50 I.U.) incubated with goat blood showed a significant decrease in percentage thrombosis (p<0.05, n = 6). Incubation of 500*μ*L goat blood with 50 I.U. resulted in only 4.4% thrombosis whilst incubation of same volume of blood with 0.05 I.U. (a 1000 times less heparin) resulted in 99.5% thrombosis. These results show that heparin has significantly potent anticoagulant properties.

**Figure 5:**
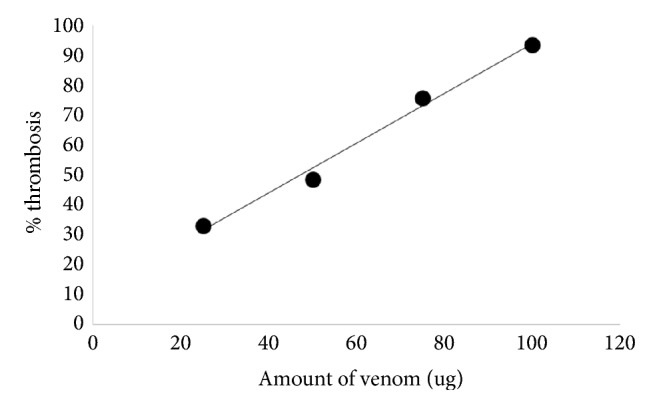
Dose-dependent coagulative (thrombotic) activity of* N. n. nigricincta* venom on Kalahari red goat blood.

As shown in Figure 5, percentage thrombosis of Kalahari red goat blood showed a very strong linear dependence on the dose of* N. n. nigricincta* venom (R^2^ = 0.9892). An increase in the amount of venom (25, 50, 75, and 100*μ*g) resulted in significantly higher levels of thrombosis (33.2%, 48.7%, 75.9%, and 93.6%, respectively). These results show that* N. n. nigricincta* venom has significantly potent procoagulant properties (p<0.05).

**Figure 6:**
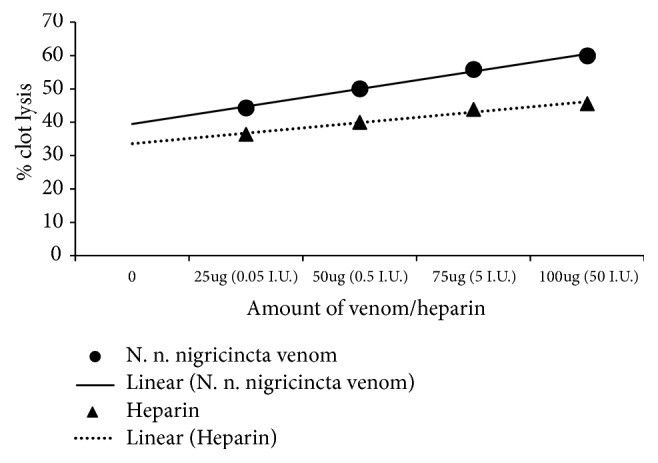
Comparison of dose-dependent thrombolytic activity of heparin and* N. n. nigricincta *venom on Kalahari red goat blood clots.

As shown in Figure 6, thrombolysis of Kalahari red goat blood clots showed very strong linear relationship with the doses of heparin (R^2^ = 0.98) and* N. n. nigricincta* (R^2^ = 0.99). At 100*μ*g* N. n. nigricincta* venom showed 60% thrombolysis which was significantly higher than the 45.6% thrombolysis in the presence of 50 I.U. heparin (p<0.05, n = 6). At 75*μ*g* N. n. nigricincta* venom showed 55.9% thrombolysis which was significantly higher than the 43.9% thrombolysis in the presence of 5 I.U. heparin (p<0.05, n = 6). At 50*μ*g* N. n. nigricincta* venom showed 50.1% thrombolysis which was significantly higher than the 40% thrombolysis in the presence of 0.5 I.U. heparin (n<0.05). At 25*μ*g* N. n. nigricincta* venom showed 44.4% thrombolysis which was significantly higher than the 36.4% thrombolysis in the presence of 0.05 I.U. heparin (p<0.05).

**Figure 7:**
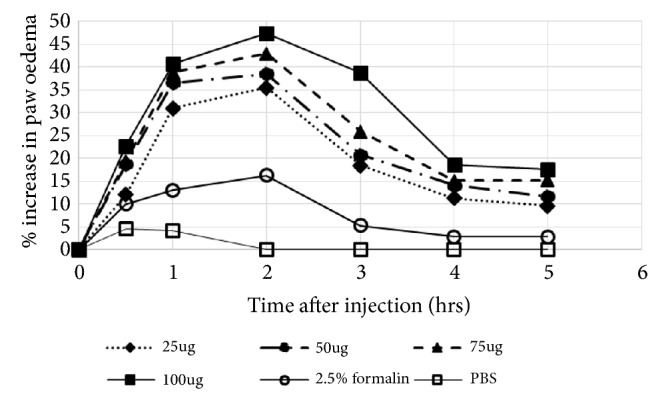
Dose-dependent oedema-forming effect of* N. n. nigricincta* venom on 10-day-old chick paw.

Subplantar injection of* N. n. nigricincta* venom into chick paw resulted in peak oedema 2-hrs after injection and subsided within 5-hrs to a mean volume ranging from 5% larger than the original volume (due to 25*μ*g venom) to 17.6% larger than the original volume (due to 100*μ*g venom) (Figure 7). The peak oedema was 35.5%, 38.5%, 42.9%, 47.5%, and 16.3% due to 25*μ*g, 50*μ*g, 75*μ*g, and 100*μ*g of venom and 2.5% formalin, respectively. Significant declines in oedema were noticed with smaller quantities of venom (100ug > 75 ug > 50 ug, respectively) (p<0.05; n=18).

Oedema due to 50*μ*g of venom was significantly greater than that due to 25*μ*g of venom which in turn was greater that oedema caused by 2.5% formalin (p<0.05). Injection of PBS resulted in a peak increase in paw size of 4.6% within the first 30mins. Paw size resolved back to normal (0% increase) within 2hrs of injection. The change in paw size due to venom injection was significantly greater than that due to PBS injection throughout the 5hrs of observation (p<0.05). The change in paw size due to 2.5% formalin was significantly greater than that due to PBS for the first 3hrs after injection (p<0.05). There was no significant difference in change in paw size between 2.5% formalin and PBS at 4hrs and 5hrs after injection (p>0.05%).

## 4. Discussion

MHD and MND have been extensively used in the preclinical assessment of viperid and crotalid venoms as important WHO approved protocols for haemorrhagic and necrotizing venom toxins. Though longstanding knowledge of cytotoxins in spitting elapids exists, the presence of powerful cytotoxins has also been well investigated and documented in nonspitting elapids. Tan and coworkers characterized a significant cytotoxin contribution to the proteomic profile of the Malayan blue coral snake [28]. The proteomic profile of the Pakistan Naja naja was also recently documented [29], though there is no documentation of MHD and MND studies on the venoms from these elapids. The assessment of haemorrhagic activity in* Micrurus pyrrhocryptus* (a Latin American elapid) venom using these protocols in mice and rats produced negative results [30]. The MHD (rabbit) of* N. n. nigricincta* venom at 9.8*μ*g was, however, almost similar to that of* Bothrops atrox*, a viper, determined using a similar assay on mice by researchers in Colombia (Otero et al., 2000). At 12.4*μ*g, the MND of* N. n. nigricincta* venom was less that the 39.3*μ*g of* Echis ocellatus* (Nigeria), 47.15*μ*g of* Echis leucogaster* (Mali), the 24.9*μ*g of* Echis pyramidum leakeyi* (Kenya), the 64.8*μ*g of* Bitis arietans* (Nigeria), and the 28.2*μ*g of* Bitis gabonica* (Nigeria) from a study by Segura et al. [31]. These findings show that* N. n. nigricincta* venom has probably successfully acquired haemorrhagic activity equal to or even surpassing those of* Viperidae*. In this study, an attempt to reduce the number of animals used by using rabbits in place of rats and mice, however, resulted in a major limitation when comparing the findings from this study to those of other earlier studies. The findings from this pioneering study with Zebra snake venom, however, provide a basis way for the use of WHO protocols involving large numbers of mice and rat for future work to determine and compare the toxicity of this venom to other

Venom from spitting elapids contains 67-73% three finger toxins (3FTXs), 22-30% phospholipases A_2_ (PLA_2_), 2.1% snake venom metalloproteinases (SVMPs), and minor quantities of nucleotidases and cysteine-rich secretory proteins (CRISPs) (Hus et al. [32]). Up to five cytotoxins (cytotoxin 1, 2, 4, 5, and 11) have been isolated and strongly implicated in the cytotoxicity of* N. mossambica*,* N annulifera,* and* N. pallida* which are all close relatives of the* N. n. nigricincta* [32]. Similar findings with another spitting cobra species,* Naja sputatrix*, revealed its venom to contain 3FTXs (64.2%), PLA_2_ (31.2%), nerve growth factor (1.82%), and SVMPs (1.33%). About 48.08% of these 3FTXs were cytotoxins [15]. In addition, PLA_2_s (acidic PLA_2_ CM I, basic PLA2 I, and basic PLA2 CMIII) have also been implicated in the cytotoxicity of* N. mossambica.* SVMPs (cobrin, atragin, and atrase) have been identified and implicated as minor contributors to cytotoxic activity in* N. atra* and* N. kaouthia* venoms [33, 34]. Isolation and identification of specific cytotoxic SVMPs in Southern African spitting elapids is, however, not yet reported. It is therefore, logical to speculate that the Southern African spitting cobras also have a smaller contribution from SVMP's towards their venoms' cytotoxicity. CRISPs (annuliferin, nawafarin, and natrin I) have been isolated and confirmed to contribute towards the cytotoxic activity of* N. nigricollis *and* N. annulifera* both of which are close relatives of the* N. n. nigricincta* [32].

The cytotoxic mechanisms of 3FTXs, PLA2s, and SVMPs mainly involve the disruption of microvascular basement membranes [35] as well as endothelial cell membranes to result in the observed haemorrhage, oedema, and myonecrosis (resulting from disruption of plasma membranes of skeletal muscle cells) [32]. These mechanisms provide possible objectives in any further investigation of the cytotoxicity of* N. n. nigricincta* venom.

*In vitro *exposure of whole goat blood to* N. n. nigricincta* venom resulted in enhancement of coagulation but the exposure of pre-formed goat blood clots resulted in profound thrombolytic activity. It is not unusual to find one venom containing both fibrinolytic (anticoagulant) and fibrinogenolytic (coagulant) activities [36, 37]. Snake venom serine proteinases (SVSPs) have been found in elapid, viperid, and colubrid venoms. These have been implicated in the interference with platelet aggregation, blood coagulation, fibrinolysis, complement system, and immune system [38]. Thrombin-like SVSPs (TLEs), however, have been implicated in procoagulation through activation of factor V, VIII, XIII, possibly VII and XI. TLEs have also been known to stimulate fibrinolysis and also activation of platelet aggregation [39]. Future studies with* N. n. nigricincta* venom can be guided towards proving or disproving involvement of these mechanisms.

An L-amino acid oxidase with human platelet aggregation activity from* Ophiophagus hannah* (king cobra) venom was isolated and characterized [40]. Cardiotoxin was isolated from* Naja naja atra* (Chinese cobra) venom; this toxin was able to potentiate platelet aggregation induced by ADP, thrombin, collagen, and venom phospholipase A_2_ [41]. Cobra venom phospholipase A_2_ showed conflicting effects on washed rabbit platelets, an initial reversible calcium-dependent aggregation followed by an inhibition of platelet aggregation with longer incubation times [42]. Two three-finger toxins, hemextin A and hemextin B, were isolated and purified from* Hemachatus haemachatus* (rinkhals) venom. Individually, hemextin A prolongs blood coagulation, but hemextin B does not show any effect on blood clotting. However, hemextin AB complex inhibits coagulation by noncompetitively inhibiting the Tissue Factor–Factor VIIa (extrinsic tenase) complex [43]. Studies to profile the proteomics of* N. n. nigricincta* venom would provide significant and relevant information that can be applied in combating envenomation from this species.

Oedema-causing toxins in snake venom have not been extensively studied. However, one study concluded that oedema induced by Bothrops snake venoms was multifactorial [44]. Other workers suggested that haemorrhagic toxins, through disruption of the microvasculature, resulted in extravasation which characterizes observed oedema in some envenomations [45, 46]. These authors also suggested the involvement of other toxins which acted directly on the endothelial cells of capillaries and venules thus increasing their permeability. Histamine release from mast cells as a result of phospholipases and cytotoxins was also a possible mechanism [47]. Another suggested mechanism was the release of prostaglandins resulting from phospholipase A_2_-induced liberation of arachidonic acid from plasma membranes [44]. In one study it was illustrated that* Bothrops jararaca* venom proteases activated plasma kininogens to bradykinin, an inflammatory mediator responsible for vasodilation (and thus oedema) at the site of inflammation [48]. Kallikrein released after vascular damage was also suggestion in the activation of kininogens to bradykinin [44]. Due to the major differences between viperid and elapids, suggestion of these mechanisms for elapid venom at best remains speculative and needs to be investigated for* N. n. nigricincta* venom.

## 5. Conclusion

In conclusion, fractionation of* N. n. nigricincta *venom and further investigations with cytotoxic, oedema-forming, procoagulant, and thrombolytic fractions separately may reveal the toxins responsible for observed activity of the venom in this study. Further work will then be required with* N. n. nigricincta* venom to unravel the mechanisms of action of any discovered toxins. The severity of the sequelae of the local cytotoxicity of this venom warrants a separate investigation into the formulation of effective intervention measures (both antivenom and other emergency on-site measures including phytotherapy) to reduce the fatalities and bodily deformities resulting from envenomation by this snake.

## Data Availability

The data used to support the findings of this study are available from the corresponding author upon request.

## References

[1] Gutiérrez J. M., Escalante T., Rucavado A., Herrera C. (2016). Hemorrhage caused by snake venom metalloproteinases: A journey of discovery and understanding. *Toxins*.

[2] Hirebail S. K., Nagabushan H., Prakash G. M. (2017). A prospective study of efficacy and safety of olopatadine versus azelastine in allergic conjunctivitis at a tertiary care hospital. *International Journal of Basic & Clinical Pharmacology*.

[3] WHO WHO guidelines for the production, control and regulation of snake antivenom immunoglobulins.

[4] Wood D., Webb C., Demeyer J. (2009). Severe snakebites in northern KwaZulu-Natal: Treatment modalities and outcomes. *South African Medical Journal*.

[5] Wagener M., Naidoo M., Aldous C. (2017). Wound infection secondary to snakebite. *South African Medical Journal*.

[6] Dalhat M. M. (2015). Socioeconomic aspects of snakebite in Africa and the tropics. *Toxinology: Clinical Toxinology in Asia Pacific and Africa*.

[7] Habib A. G., Brown N. I. (2018). The snakebite problem and antivenom crisis from a health-economic perspective. *Toxicon*.

[8] White J., Meier J. Handbook of clinical toxicology of animal venoms and poisons.

[9] Albaba I. (2017). Venomous snakes and envenomation in Palestine. *Journal of Entomology and Zoology Studies*.

[10] Choudhury M., McCleary R. J. R., Kesherwani M., Kini R. M., Velmurugan D. (2017). Comparison of proteomic profiles of the venoms of two of the ‘Big Four’ snakes of India, the Indian cobra (Naja naja) and the common krait (Bungarus caeruleus), and analyses of their toxins. *Toxicon*.

[11] Sharma N., Chauhan S., Faruqi S., Bhat P., Varma S. (2005). Snake envenomation in a north Indian hospital. *Emergency Medicine Journal*.

[12] Upadhyay U., Upadhyay U., Mohanty N. K., Behera S. K. (2017). Neurotoxic snakebite cases with role of imaging. *Journal of Evidence Based Medicine and Healthcare*.

[13] Tan C. H., Tan K. Y., Lim S. E., Tan N. H. (2015). Venomics of the beaked sea snake, Hydrophis schistosus: A minimalist toxin arsenal and its cross-neutralization by heterologous antivenoms. *Journal of Proteomics*.

[14] Wong K. Y., Tan C. H., Tan N. H. (2016). Venom and purified toxins of the spectacled cobra (Naja naja) from Pakistan: Insights into toxicity and antivenom neutralization. *The American Journal of Tropical Medicine and Hygiene*.

[15] Tan C. H., Wong K. Y., Tan K. Y., Tan N. H. (2017). Venom proteome of the yellow-lipped sea krait, Laticauda colubrina from Bali: Insights into subvenomic diversity, venom antigenicity and cross-neutralization by antivenom. *Journal of Proteomics*.

[16] Goswami P. K., Samant M., Srivastava R. S. (2014). Snake venom, anti-snake venom & potential of snake venom. *International Journal of Pharmacy and Pharmaceutical Sciences*.

[17] Alirol E., Sharma S. K., Bawaskar H. S., Kuch U., Chappuis F. (2010). Snake bite in south asia: a review. *PLOS Neglected Tropical Diseases*.

[18] De Vries A., Cohen I. (1969). Hemorrhagic and blood coagulation disturbing action of snake venoms. *Recent Advances in Blood Coagulation*.

[19] Tan K. Y., Tan N. H., Tan C. H. (2018). Venom proteomics and antivenom neutralization for the Chinese eastern Russell’s viper, Daboia siamensis from Guangxi and Taiwan. *Scientific Reports*.

[20] Wüster W., Crookes S., Ineich I. (2007). The phylogeny of cobras inferred from mitochondrial DNA sequences: Evolution of venom spitting and the phylogeography of the African spitting cobras (Serpentes: Elapidae: Naja nigricollis complex). *Molecular Phylogenetics and Evolution*.

[21] Félix-Silva J., Silva-Junior A. A., Zucolotto S. M., de Freitas Fernandes Pedrosa M. (2017). Medicinal plants for the treatment of local tissue damage induced by snake venoms: an overview from traditional use to pharmacological evidence. *Evidence-Based Complementary and Alternative Medicine*.

[22] Gasanov S. E., Dagda R. K., Rael E. D. (2014). Snake Venom Cytotoxins, Phospholipase A2s, and Zn2+-dependent Metalloproteinases: Mechanisms of Action and Pharmacological Relevance. *Journal of Clinical Toxicology*.

[23] Stewart R. M., Page C. P., Schwesinger W. H., McCarter R., Martinez J., Aust J. B. (1989). Antivenin and fasciotomy/debridement in the treatment of the severe rattlesnake bite. *The American Journal of Surgery*.

[24] WHO WHO Guidelines for the Production, Control and Regulation of Snake Antivenom Immunoglobulins.

[25] Kondo H., Kondo S., Ikezawa H., Murata R. (1960). Studies on the quantitative method for determination of hemorrhagic activity of habu snake venom. *Japanese Journal of Medical Science and Biology*.

[26] Fereidoni M., Ahmadiani A., Semnanian S., Javan M. (2000). An accurate and simple method for measurement of paw edema. *Journal of Pharmacological and Toxicological Methods*.

[27] Ainooson G. K., Owusu G., Woode E., Ansah C., Annan K. (2012). Trichilia monadelpha bark extracts inhibit carrageenan-induced foot-oedema in the 7-day old chick and the oedema associated with adjuvant-induced arthritis in rats. *African Journal of Traditional, Complementary, and Alternative Medicines : AJTCAM/African Networks on Ethnomedicines*.

[28] Tan C. H., Fung S. Y., Yap M. K. K., Leong P. K., Liew J. L., Tan N. H. (2016). Unveiling the elusive and exotic: Venomics of the Malayan blue coral snake (Calliophis bivirgata flaviceps). *Journal of Proteomics*.

[29] Wong K. Y., Tan C. H., Tan K. Y., Quraishi N. H., Tan N. H. (2018). Elucidating the biogeographical variation of the venom of Naja naja (spectacled cobra) from Pakistan through a venom-decomplexing proteomic study. *Journal of Proteomics*.

[30] Dokmetjian J. C., del Canto S., Vinzón S., de Jiménez Bonino M. B. (2009). Biochemical characterization of the Micrurus pyrrhocryptus venom. *Toxicon*.

[31] Segura Á., Villalta M., Herrera M. (2010). Preclinical assessment of the efficacy of a new antivenom (EchiTAb-Plus-ICP®) for the treatment of viper envenoming in sub-Saharan Africa. *Toxicon*.

[32] Hus K., Buczkowicz J., Petrilla V. (2018). First look at the venom of naja ashei. *Molecules*.

[33] Tan N. H., Wong K. Y., Tan C. H. (2017). Venomics of Naja sputatrix, the Javan spitting cobra: A short neurotoxin-driven venom needing improved antivenom neutralization. *Journal of Proteomics*.

[34] Huang H.-W., Liu B.-S., Chien K.-Y. (2015). Cobra venom proteome and glycome determined from individual snakes of Naja atra reveal medically important dynamic range and systematic geographic variation. *Journal of Proteomics*.

[35] G. Konshina A., V. Dubovskii P., G. Efremov R. (2012). Structure and dynamics of cardiotoxins. *Current Protein & Peptide Science*.

[36] Oliveira C. H., Simão A. A., Trento M. V., César P. H., Marcussi S. (2016). Inhibition of proteases and phospholipases A2 from Bothrops atrox and Crotalus durissus terrificus snake venoms by ascorbic acid, vitamin E, and B-complex vitamins. *Anais da Academia Brasileira de Ciências*.

[37] Matsui T., Fujimura Y., Titani K. (2000). Snake venom proteases a í ecting hemostasis and thrombosis. *Biochimica et Biophysica Acta (BBA)*.

[38] Kini R. M. (2006). Serine proteases affecting blood coagulation and fibrinolysis from snake venoms. *Pathophysiology of Haemostasis and Thrombosis*.

[39] Sajevic T., Leonardi A., Križaj I. (2011). Haemostatically active proteins in snake venoms. *Toxicon*.

[40] Li Z.-Y., Yu T.-F., Lian E. C.-Y. (1994). Purification and characterization of l-amino acid oxidase from king cobra (Ophiophagus hannah) venom and its effects on human platelet aggregation. *Toxicon*.

[41] Teng C.-M., Jy W., Ouyang C. (1984). Cardiotoxin from Naja naja atra snake venom: A potentiator of platelet aggregation. *Toxicon*.

[42] Teng C.-P., Kuo Y.-P., Lee L.-G., Ouyang C. (1986). Effect of cobra venom phospholipase A2 on platelet aggregation in comparison with those produced by arachidonrc acid and lysophophatidylcholine. *Thrombosis Research*.

[43] Banerjee Y., Lakshminarayanan R., Vivekanandan S., Anand G. S., Valiyaveettil S., Kini R. M. (2007). Biophysical characterization of anticoagulant hemextin AB complex from the venom of snake Hemachatus haemachatus. *Biophysical Journal*.

[44] Gutiérrez J. M., Lomonte B., Gutiérrez J. M. (1989). Local tissue damage induced by Bothrops snake venoms - A review. *Memórias do Inst. Butantan*.

[45] Ownby C. L. (1982). Pathology of rattlesnake envenomation. *Rattlesnake Venoms*.

[46] Ohsaka A., Lee C.-Y. (1979). Hemorrhagic, Necrotizing and Edema-Forming Effects of Snake Venoms. *Snake Venoms*.

[47] Preciado L., Pereañez J. A., Nuñez V., Lobo-Echeverri T. (2016). Characterization of the most promising fraction of Swietenia macrophylla active against myotoxic phospholipases A2: Identification of catechin as one of the active compounds. *Vitae*.

[48] Rocha e silva M., Beraldo W. T., Rosenfeld G. (1949). Bradykinin, a hypotensive and smooth muscle stimulating factor released from plasma globulin by snake venoms and by trypsin. *American Journal of Physiology-Endocrinology and Metabolism*.

